# Interactive effects of combined manganese and microplastic pollution on soybean growth

**DOI:** 10.3389/fpls.2025.1699129

**Published:** 2025-11-18

**Authors:** Jun Ren, Xuqiang Luo, Yang Luo, Xiuyue Xu, Qian Wang, Qifang Zhang

**Affiliations:** School of Geography and Resources, Guizhou Education University, Guiyang, China

**Keywords:** microplastics, manganese toxicity, combined pollution, antioxidant enzymes, soybean biomass accumulation

## Abstract

Soil microplastics (MPs) and heavy metals pose a threat to agricultural production. Excessive manganese (Mn) can be toxic to plants. The impacts of combined MP and Mn pollution in soil on plant growth are poorly understood. This pot experiment systematically investigated the effects of single or combined pollution by polyethylene microplastics (PE-MPs) at different concentrations (0%, 0.50%, and 1.5%) and Mn (MnSO_4_, 0%, 0.25%, and 0.50%) on soybean growth. The addition of Mn + PE-MPs significantly reduced soil pH; PE-MPs increased soil organic matter (OM), while Mn (0.25%) + PE-MPs significantly decreased soil OM. Both single and combined additions of Mn and PE-MPs significantly reduced soil available nitrogen and available phosphorus, but increased soil available potassium. Polyethylene microplastics decreased the number of soybean root tips, whereas Mn (0.25%) resulted in a significant increase. Soybean biomass was highest under the Mn (0.25%) + PE-MPs (0.50%) treatment. PE-MPs significantly increased the peroxidase, catalase, and superoxide dismutase activities in soybeans, while Mn (0.25%) significantly reduced their activities. The addition of low concentrations of Mn alleviated the PE-MP stress on soybeans and exerted a detoxifying effect. These findings provide scientific support for the safe production of soybeans.

## 1 Introduction

Microplastics (MPs) are plastic particles with a diameter of less than 5 mm. Common MPs found in farmland include polyethylene (PE), polypropylene (PP), and polystyrene (PS) (Li et al., 2024b). Polyethylene accounts for more than 40% of the detected MPs in agricultural soils, with residual agricultural mulch films being the primary source of polyethylene microplastics (PE-MPs) (Bai et al., 2024). As the most abundant plastic pollutant residue in farmland ecosystems, the environmental behaviors and ecological effects of PE-MPs pose a significant threat to the sustainable development of global agriculture. Microplastics are regarded as emerging pollutants in ecosystems (Wang et al., 2024a). Polyethylene MPs directly damage soil health by altering pore structure and water movement. When their concentration is ≥1%, soil porosity decreases by 15%, and the stability of large aggregates declines, leading to increasing resistance to the extension of plant roots (Dan et al., 2025). Exposure to PE-MPs reduces the xylem hydraulic conductivity of cabbage by 86.8%, thereby indirectly inhibiting plant growth (Yu et al., 2022). The inhibitory effects of MPs on plant growth also vary at different MP doses (Zhang et al., 2022). Polyethylene MPs can disrupt the balance of soil elements through the dynamic process of surface adsorption-desorption. The oxygen-containing functional groups formed during PM-MP aging can adsorb heavy metal ions, prolonging the bioavailability of heavy metals in the rhizosphere (Ma et al., 2023; Iqbal et al., 2025). The exposure dose of MPs affects their interaction with heavy metals, and MPs can reduce the adsorption capacity of soil for cadmium (Cd) (Wang et al., 2020b, Wang et al., 2024b).

In the agricultural field system, the co-pollution of MPs and heavy metals (HMs) is particularly prevalent (Zhou et al., 2021). MPs in the soil can accelerate the migration rate of HMs in the soil, thus exacerbating the hazards posed by single-pollutant contamination (Medyńska-Juraszek and Jadhav, 2022). Some studies have revealed that MPs are capable of adsorbing substantial amounts of HMs in the soil, including arsenic (As), cadmium (Cd), chromium (Cr), copper (Cu), nickel (Ni), lead (Pb), and zinc (Zn) (Abbasi et al., 2020; Sarkar et al., 2021). They serve as transport vectors for the migration of HMs. Once reaching a certain concentration, MPs disperse HMs into the soil through the process of desorption. The pollution of MPs and HMs can remarkably impact the physicochemical properties of soil. Evidences from relevant research indicate that the sole addition of Cd may result in a decline in the soil pH, whereas the addition of polyethylene (PE) can give rise to an elevation in the pH (Wang et al., 2020a). The combined pollution of MPs and HMs exerts a remarkable inhibitory effect on soil organic matter, which is mainly attributed to its impact on the changes in the soil microbial community (Dong et al., 2021). The metabolic effects of the combined pollution on plant growth are achieved by influencing the physicochemical properties of the soil and the plants’ own physiological responses (Ziani et al., 2023). Studies have shown that the combined pollution of polyvinyl chloride (PVC) and Cr significantly reduced the biomass of sweet potatoes, and the combined pollution of PVC and Cd significantly damaged the photosynthetic system of cucumber seedlings (Li et al., 2020; Khan et al., 2023). As an essential trace element for plant growth, Mn is a key component of plant chlorophyll and plays an important role in photosynthesis and the activation of antioxidant enzymes. It is involved in plant metabolic processes, including oxygenic photosynthesis, respiration, and enzyme activation (Wang et al., 2023). Plants require only 20−40 mg/kg (dry weight) of Mn to sustain normal growth. An excessive amount of Mn can lead to plant toxicity. In many regions worldwide, Mn availability is a key limiting factor for crop productivity (Hou et al., 2022). When soybeans are subjected to Mn toxicity stress, their cellular defense mechanisms are activated, and the activities of corresponding antioxidant enzymes increase accordingly. In high-Mn environments, soybeans exhibit a variety of symptoms, including a reduction in root biomass and the inhibition of root development (Liu et al., 2023). The influence of Mn on plant growth is different from that of other HMs. Under the condition of combined pollution of MPs and Mn, the soil physical and chemical properties and plant growth status are a valuable research topic.

Most previous studies of soybean growth focused on a single pollutant (MPs or Mn) (El Amine et al., 2023; Rong et al., 2023; Lo Porto et al., 2024). The interactive effects of PE−MPs and Mn combined pollution on the growth phenotype, root system, and antioxidant enzymes of soybeans have been ignored. This study investigated the growth characteristics of soybean seedlings under combined pollution scenarios with different doses of PE−MPs and Mn. This study revealed the characteristics of changes in the root system, biomass, and antioxidant enzyme activities of soybeans under combined pollution. This research provides scientific support for the safe production of soybeans.

## 2 Materials and methods

### 2.1 Pot experiment design

Polyethylene MP particles with a diameter of 100 μm were procured from Ningbo Lanting Microplastics Co., Ltd., China. Manganese sulfate (MnSO_4_) was purchased from Xi’an Kalang Trading Co., Ltd., China. The soil was collected from farmland near Guizhou Education University. After being naturally air-dried, large stones and visible impurities in the soil were removed. Soybeans were grown using the pot culture method. The experiment was conducted in a greenhouse at Guizhou Education University in May 2025. Different proportions of PE were mixed into the soil. Manganese sulfate solutions of different concentrations were then prepared and poured into the soil, followed by thorough mixing. Soil with no added substances served as the control (CK). Plastic pots with specifications of 25 cm diameter × 17.5 cm height were used to hold 3 kg of mixed substrate. The detailed treatment ratios are shown in **Table 1**. Each treatment had three replicates. The water content of the substrate was adjusted to approximately 60% of the field capacity by the weighing method, and the pots were then stabilized at room temperature for 14 days. Soybean seeds (Zhonghuang 13) were purchased from Qingxian Chunfeng Seed Co., Ltd., China. Uniform-sized and plump soybean seeds were selected for the experiment, with 30 seeds sown in each pot. During the seedling growth period, the soil water-holding capacity was maintained at around 60%. The phenotypic and physiological indices of the seedlings were determined 30 days after planting.

**Table 1:** Scheme of experimental design.

Treatment	PE-MPs (%)	MnSO_4_ (%)
CK	0	0
T1	0.50	0
T2	1.50	0
T3	0	0.25
T4	0.50	0.25
T5	1.50	0.25
T6	0	0.50
T7	0.50	0.50
T8	1.50	0.50

The addition of pollutants is based on the mass ratio to the soil.

### 2.2 Germination rate and plant height

Starting from the first day of germination, the cumulative number of germinated seeds was counted daily, and the counting ended on the 7th day. The germination rate could then be determined as (number of normally germinated seeds/total number of tested seeds) × 100%. The height of soybean plants was measured with a tape measure. The average value of five randomly selected plants per pot was taken as the plant height data.

### 2.3 Determination of physiological indices

Chlorophyll was extracted with 95% ethanol, and the absorbance was measured at 649 nm and 665 nm (Luo et al., 2024). The chlorophyll a, chlorophyll b, and total chlorophyll contents were calculated. The malondialdehyde (MDA) content was determined using a plant MDA assay kit based on the thiobarbituric acid (TBA) method. The peroxidase (POD) activity was measured using a detection kit based on the guaiacol colorimetric method; the superoxide dismutase (SOD) activity was determined with a detection kit based on the nitroblue tetrazolium (NBT) riboflavin colorimetric method; and the catalase (CAT) activity was assayed by a detection kit based on the ultraviolet colorimetric method. These kits were purchased from Shanghai Yuanye Biotechnology Co., Ltd. To reduce errors, the samples required for determining each index were collected from the same horizontal position of the plants.

### 2.4 Determination of biomass and root morphology

The aboveground parts of all plants in each pot were cut off, then rinsed with tap water, cleaned with deionized water, blotted dry, and their fresh weight was measured using a balance. To assess the growth status of plant root systems, roots were carefully separated to keep them intact. One complete root system was randomly selected from each pot, and loosely attached soil was washed off with tap water, followed by rinsing with distilled water. The cleaned root was placed in a transparent tray with an even layer of distilled water and scanned using a root nodule assessment system (GXY-A1 root analyzer, Top Instrument, Zhejiang, China). The scanned images were analyzed with RootAnalysis Pro software to measure root morphological parameters, including the number of root tips, total length (mm), root diameter (mm), and total surface area. The scanned roots and root systems within the same pot were placed into kraft paper bags. Along with the above-ground parts (after fresh weight had been measured), they were subjected to a 30-minute fixation treatment at 105 °C. Samples were then dried at 65 °C until a constant weight was achieved. The stem, root, and total biomass were recorded. The dried samples were crushed and stored for the determination of Mn concentrations. When harvesting the root systems, rhizosphere soil was collected simultaneously. First, the soil loosely attached to the roots was gently shaken off, then the soil within 5 mm of the root surface was brushed off using a sterile soft brush. After being naturally air-dried, the collected rhizosphere soil was used to determine soil pH and nutrient indices.

### 2.5 Soil pH and nutrient indices

After harvesting the plants, part of the soil was collected, spread out, and naturally air-dried. The air-dried soil samples were ground and passed through a sieve with a 2-mm aperture, followed by thorough mixing. This soil was used for a pH determination using a pH meter. The remaining naturally air-dried soil was passed through a 0.85 mm sieve for the determination of soil nutrient indicators. The available nitrogen (AN) in the soil was determined by the alkali-hydrolysis diffusion method (Camargo et al., 1997); available phosphorus (AP) was measured by the NaHCO_3_ extraction-molybdenum antimony anti-colorimetric method; available potassium (AK) was analyzed by the ammonium acetate extraction-flame atomic absorption method (Ge et al., 2018); and the organic matter (OM) content was determined by the potassium dichromate external heating method (Strickland and Sollins, 1987).

### 2.6 Determination of Mn in soil and plants

Determination of soil Mn: The soil was naturally air-dried, ground, and passed through a 100-mesh nylon sieve. A 0.1 g sample was placed in a polytetrafluoroethylene digestion tank, then 3 mL of nitric acid and 1 mL of hydrofluoric acid were added. After digestion in a drying oven at 180°C for 20 h, 1 mL of perchloric acid was added, and the mixture was digested on an electric hot plate at 200°C for 2 h. The lid was opened to drive off the acid until it was nearly dry. Then, 1 mL of nitric acid was added to dissolve the residue, and the volume was made up to 50 mL with ultrapure water for testing.

Determination of plant Mn: A 0.2 g sample of dried and crushed plant material was weighed and placed in a polytetrafluoroethylene digestion tank. Then, 3 mL of nitric acid was added, and after digestion in a drying oven at 160°C for 18 h, the mixture was digested on an electric hot plate at 200°C for 2 h. The lid was opened to drive off the acid until it was nearly dry. Finally, 1 mL of nitric acid was added to dissolve the residue, and the volume was made up to 25 mL with ultrapure water for testing.

An inductively coupled plasma mass spectrometer (ICP-MS-XII, Thermo Fisher Scientific, Waltham, MA, USA) was used to analyze the above solutions (Bressy et al., 2013). During the entire experimental process, standard substances (GSS series for soil, GSV series for plants), blank controls, and parallel tests were used for quality control. All reagents were ultra-high purity grade (Luo et al., 2024).

### 2.7 Statistical analysis

All data was analyzed using Excel. Various parameters, including germination rate, plant height, plant antioxidant enzyme activity, biomass, root morphology, soil nutrient indices, and plant Mn uptake characteristics under different treatments, were calculated and presented as the mean ± standard deviation (SD). Results were visualized as bar charts using Origin 2021. A one-way ANOVA was conducted using IBM SPSS Statistics 26.0 to evaluate differences among treatments. Multiple comparisons were conducted using the Least Significant Difference (LSD) test. Additionally, mantel tests and correlation heatmap analyses were conducted using R4.4.2 software to explore the relationships between variables.

## 3 Results

### 3.1 Effects of Mn and PE-MPs on soil nutrients

**Figure 1:**
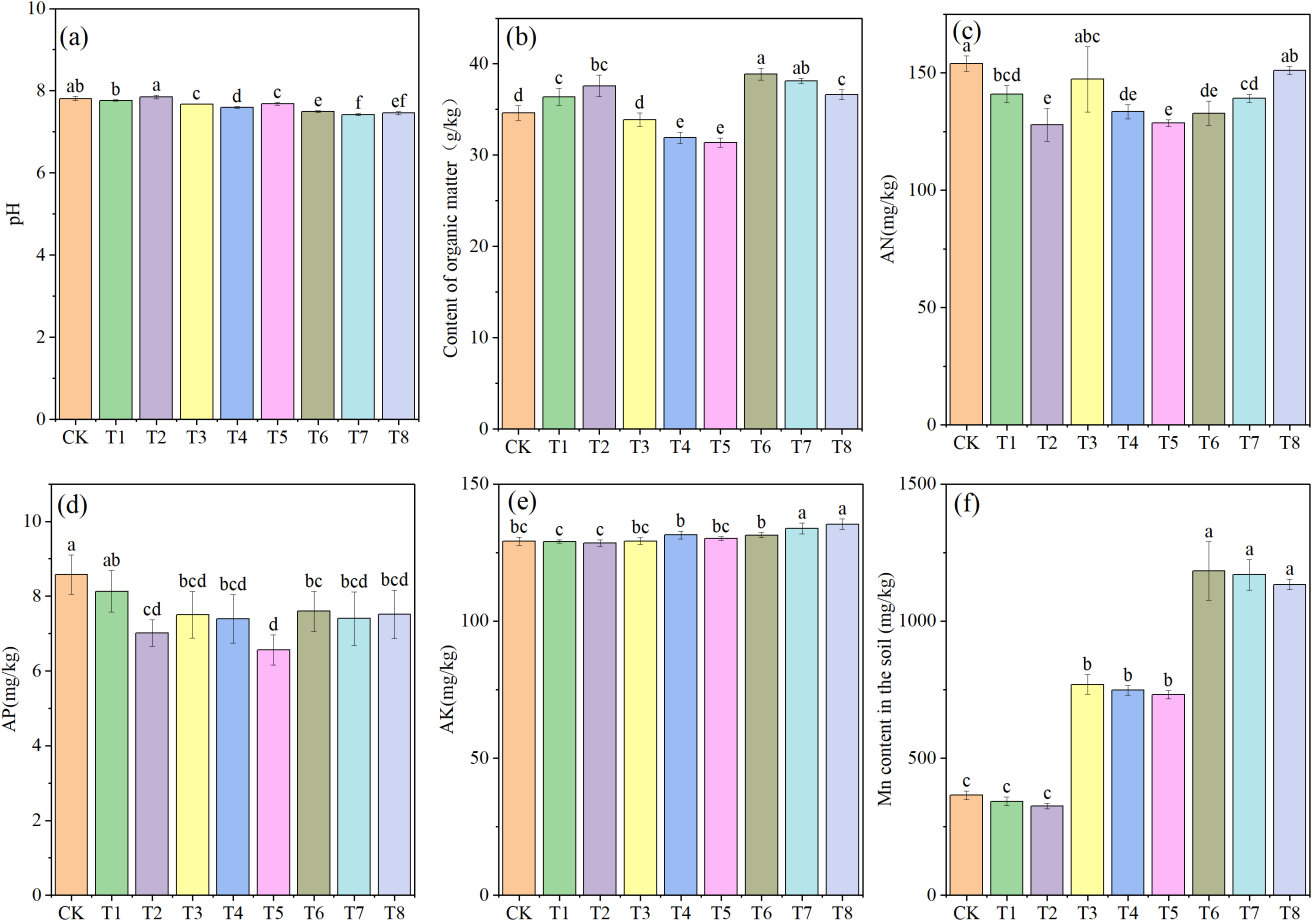
Effects of combined Mn and PE-MPs pollution on key soil nutrient parameters: **(A)** pH, **(B)** organic matter, **(C)** AN: available nitrogen, **(D)** AP: available phosphorus, **(E)** AK: available potassium, and **(F)** MnS: manganese concentration in soil. Different lowercase letters indicate significant differences among the different treatments (*P* < 0.05).

**Figure 1** shows the soil nutrient indices across different treatments. The soil pH ranged from 7.42 to 7.85. Compared with the CK, Mn application significantly reduced soil pH (*P* < 0.05) in the 0.25% and 0.5% treatments. In contrast, the addition of PE-MPs had no noticeable effect on pH. The lowest soil pH was recorded under the combined treatment of 1.5% PE-MPs and 0.50% Mn. Soil OM content also displayed distinct variations, with PE-MPs alone significantly increasing the OM content (*P* < 0.05). However, in the 0.25% and 0.50% Mn treatments, the addition of PE-MPs significantly reduced the OM content (*P* < 0.05). Notably, among all treatments, the three with an Mn addition level of 0.25% had the lowest OM content, with decreases of 2.11%, 7.80%, and 9.36% for T3, T4, and T5, respectively. The application of Mn significantly reduced the soil AN content (*P* < 0.05). The addition of PE-MPs alone also significantly lowered the AN content (*P* < 0.05), and their co-application with Mn resulted in a more pronounced decrease (*P* < 0.05), with the lowest AN content observed under the 0.25% Mn + 1.5% PE-MPs treatment. Soil AP ranged from 6.57 to 8.58 mg/kg. Both PE-MPs and Mn individually caused significant reductions in the soil AP content (*P* < 0.05), and their combined effect further intensified this decrease (*P* < 0.05). The lowest AP content, representing a 23.42% reduction compared to the CK, occurred with the 0.25% and 1.5% treatments. The soil AK content ranged from 128.48 to 135.37 mg/kg. Both the addition of Mn and PE-MPs increased the AK content in the soil. The highest AK content was observed under the 0.50% and 1.5% treatments, which both produced a significant increase (*P* < 0.05). Following Mn application, the total soil Mn concentration ranged from 325.11 to 1183.14 mg/kg.

### 3.2 Effects of Mn and PE-MPs on soybean growth

**Figure 2:**
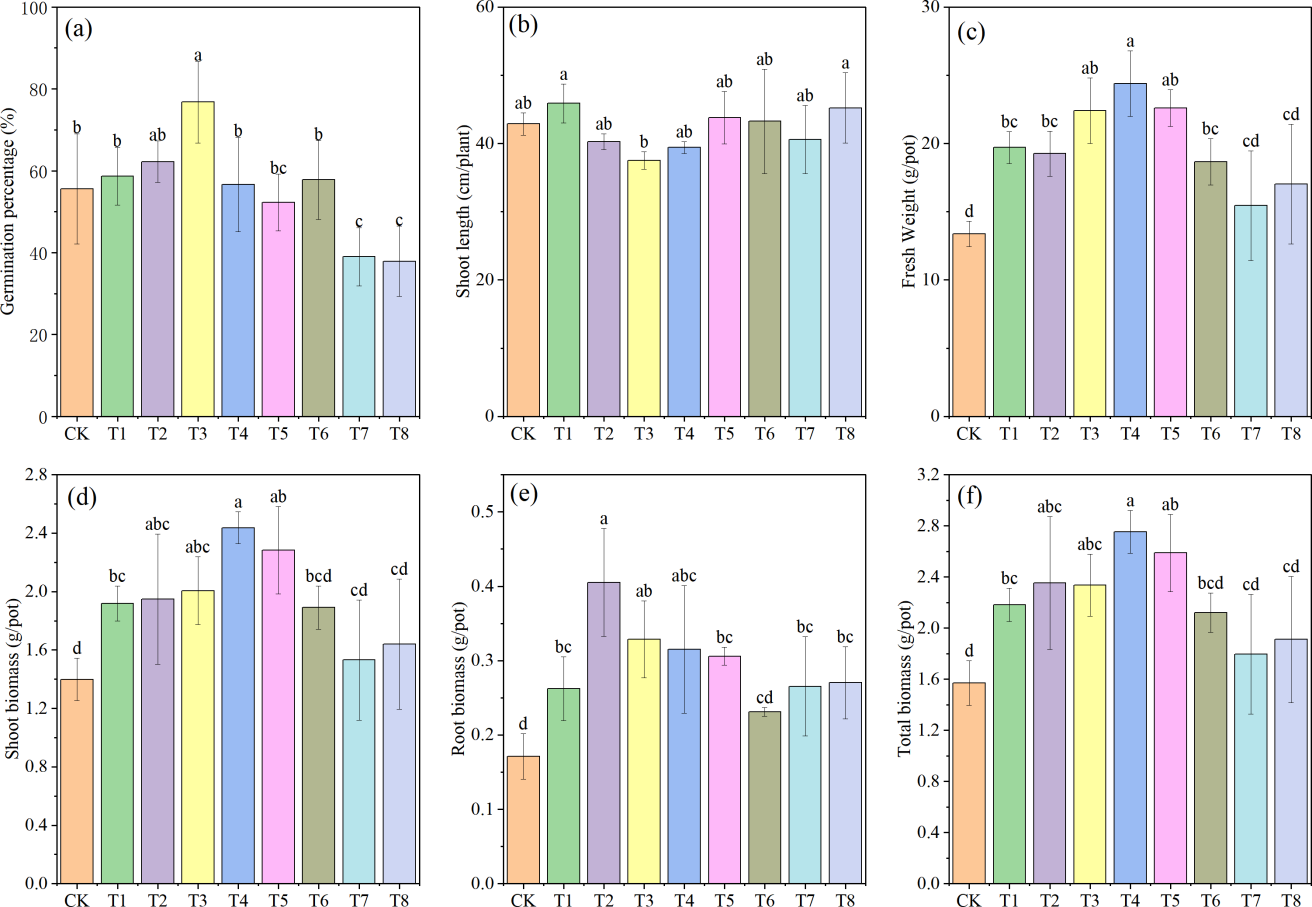
Effects of combined Mn and PE-MPs pollution on soybean growth parameters: **(A)** germination percentage, **(B)** shoot length, **(C)** fresh Weight, **(D)** root biomass, **(E)** shoot biomass, and **(F)** total biomass. Different lowercase letters indicate significant differences among the different treatments (*P* < 0.05).

Determinations of germination rate, plant height, fresh weight, and biomass revealed significant differences among the various treatments. As shown in **Figure 2**, compared with the CK group, both the individual addition of Mn and PE-MPs enhanced the germination rate of soybeans. The highest germination rate was observed in the 1.5% PE-MPs treatment, with an 11.98% increase, although this increase was not statistically significant. At 0.25% Mn, the germination rate reached 76.78%, a significant increase of 38.19% (*P* < 0.05). In contrast, the combined addition of Mn and PE-MPs inhibited soybean germination; significant inhibition was observed when Mn was applied at 0.50% combined with PE-MPs at either 0.5% or 1.5% (*P* < 0.05). The addition of Mn and PE-MPs had no significant impact on plant height. However, their effects on biomass and fresh weight were notable, with both substances significantly increasing soybean biomass (*P* < 0.05). The three treatments with 0.25% Mn exhibited a relatively high biomass, with the maximum biomass (2.75 g) recorded under the 0.5% PE-MPs treatment, representing an 81.57% increase over the CK. The trends in soybean fresh weight and stem-leaf biomass were consistent with those of total biomass.

### 3.3 Effects of Mn and PE-MPs on root morphology

**Figure 3:**
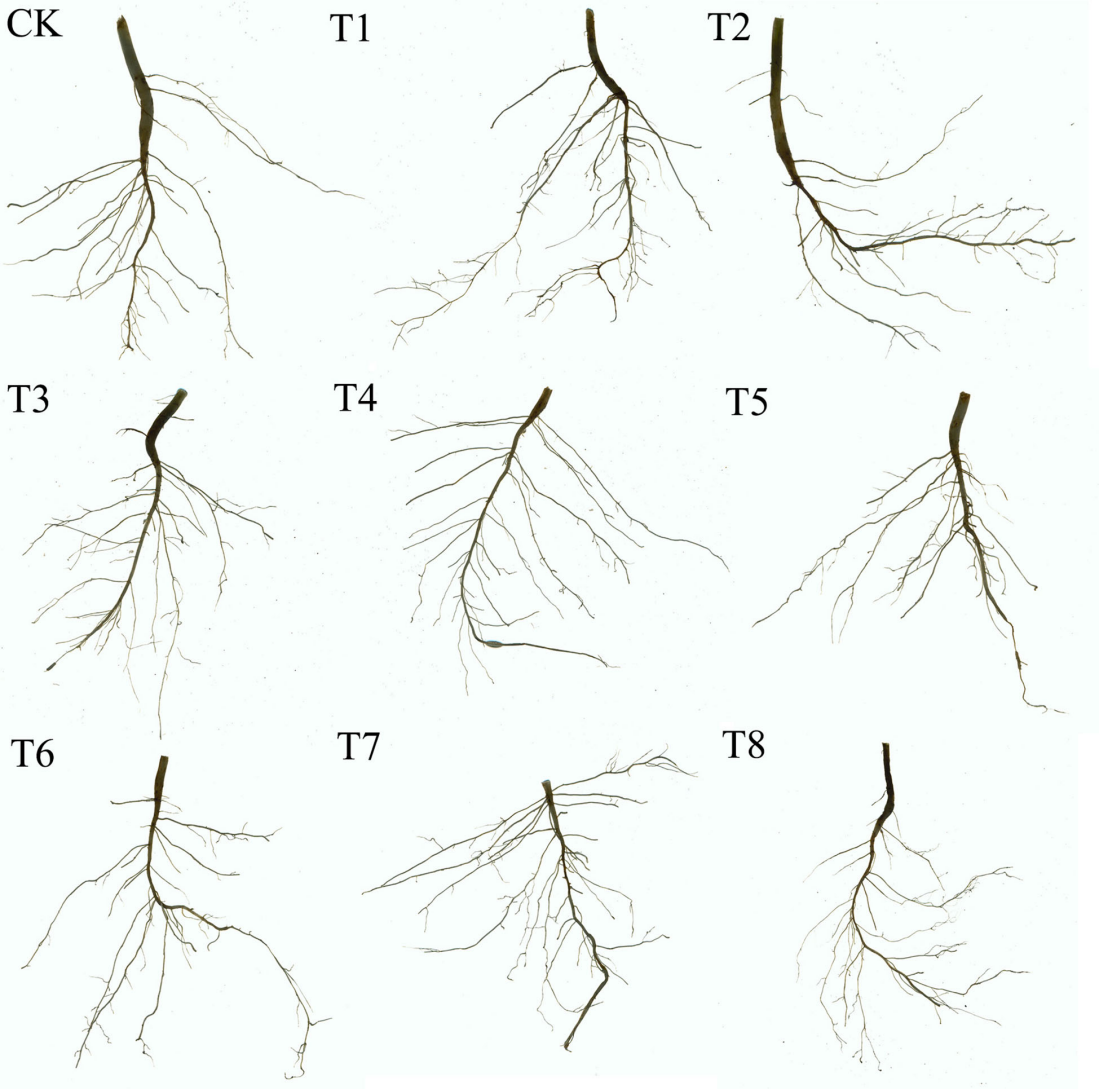
The morphology of soybean roots under combined Mn and PE-MPs pollution.

**Figure 4:**
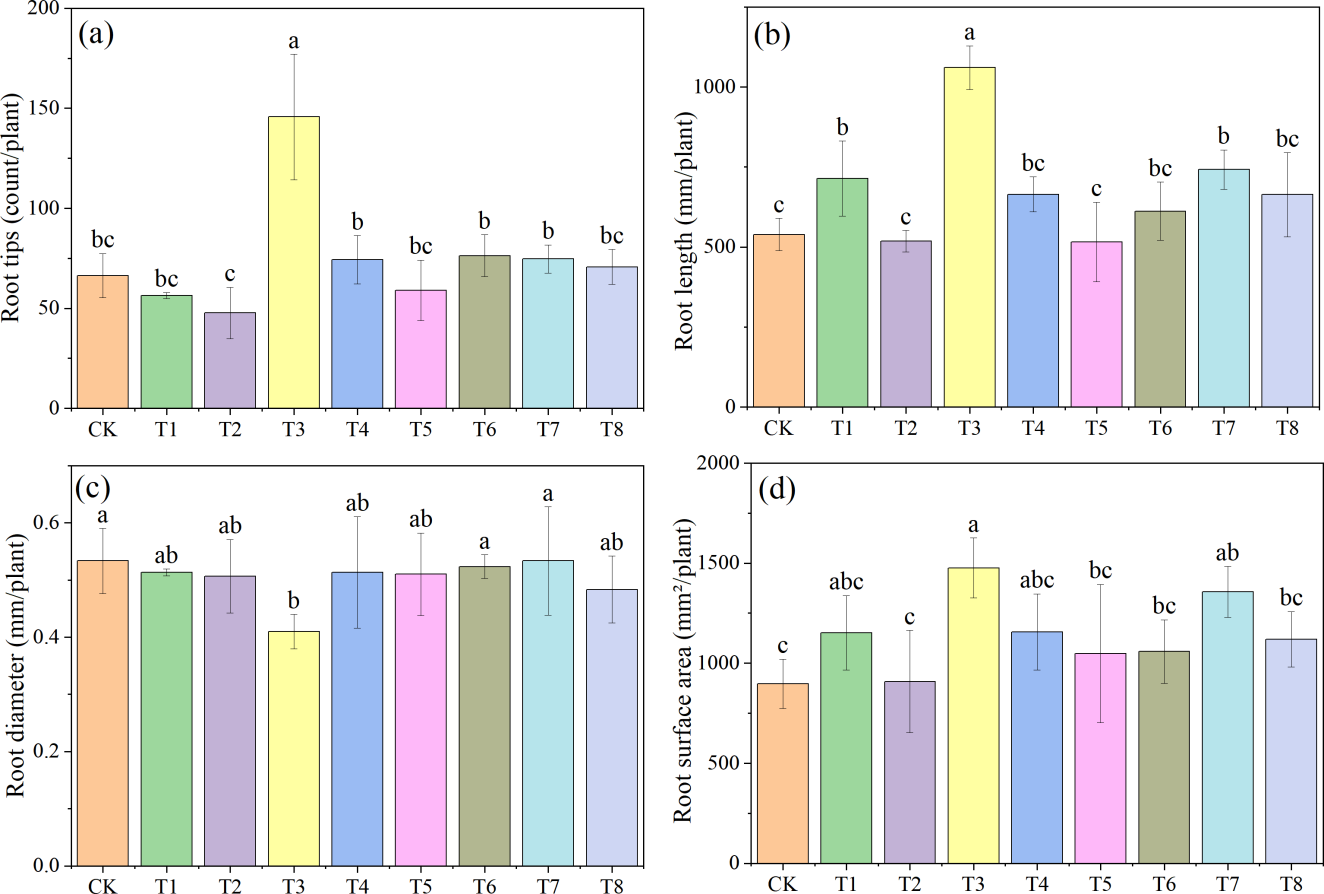
Effects of combined Mn and PE-MPs pollution on soybean root morphological traits: **(A)** root tips, **(B)** root length, **(C)** root diameter, and **(D)** root surface area. Different lowercase letters indicate significant differences among the different treatments (*P* < 0.05).

**Figure 3** shows the morphological characteristics of soybean roots under different treatments (one representative pot from each treatment was photographed). **Figure 4** presents the effects of combined applications of Mn and PE-MPs on soybean root morphology, with significant differences observed in the number of root tips, total root length, root diameter, and total root surface area among the different treatments. The results show that the number of soybean root tips ranged from 47 to 145. Compared with the CK, PE-MPs reduced the number of root tips, with a significant reduction observed under the 1.5% treatment (*P* < 0.05). The addition of Mn increased the number of root tips, with a significant increase under the 0.25% treatment (*P* < 0.05), while no increase was apparent at 0.50%. The combined addition of Mn and PE-MPs had no significant effect on root tips. The addition of PE-MPs also increased the total root length, with a significant increase under the 0.5% treatment (*P* < 0.05), while no effect was apparent at 1.5%. The combined addition of Mn and PE-MPs increased the total root length, but the increase was not significant. The effect of Mn and PE-MPs on the total root surface area followed a trend consistent with that for total root length. The combined addition of PE-MPs and Mn had no obvious effect on root diameter. However, when only 0.25% Mn was added, the root diameter was the smallest and was significantly different from the CK (*P* < 0.05).

### 3.4 Effects of Mn and PE-MPs on chlorophyll content and antioxidant enzyme activities in soybeans

**Figure 5:**
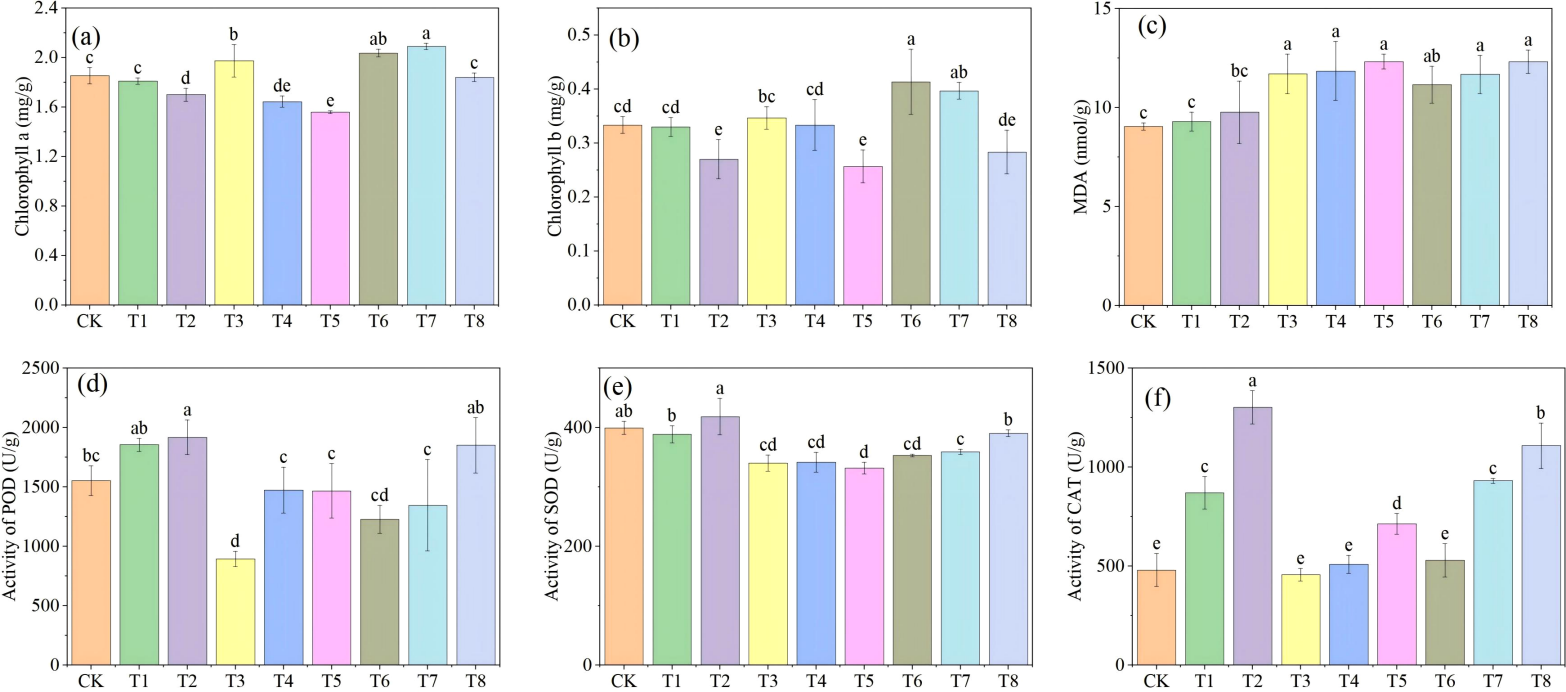
The effects of combined Mn and PE-MPs pollution on soybean chlorophyll, MDA, and antioxidant enzymes: **(A)** chlorophyll a, **(B)** chlorophyll b, **(C)** MDA, **(D)** POD, **(E)** SOD, **(F)** CAT. Different lowercase letters indicate significant differences among the different treatments (*P* < 0.05).

The impacts of Mn and PE-MPs on soybeans were assessed by measuring chlorophyll content, MDA levels, and the activities of antioxidant enzymes (POD, SOD, and CAT) in soybean leaves (**Figure 5**). The results indicated that, relative to the CK, Mn significantly elevated leaf chlorophyll a content (*P* < 0.05), with the maximum increase observed under the 0.50% treatment. In contrast, PE-MPs reduced leaf chlorophyll a content, with a significant decrease under the 1.5% treatment (*P* < 0.05). The co-application of Mn and PE-MPs had no significant effect on chlorophyll a. The influence of Mn and PE-MPs on total chlorophyll in soybeans followed a trend consistent with that of chlorophyll a. The application of Mn significantly increased the soybean MDA content (*P* < 0.05), peaking at 11.71 nmol/g under the 0.25% treatment. The addition of PE-MPs also induced an increase in MDA content, although this effect was not statistically significant. The co-application of Mn and PE-MPs led to a significant elevation in MDA content (*P* < 0.05), with the highest value recorded under the 0.50% Mn+1.5% PE-MPs treatment. The addition of PE-MPs significantly enhanced the POD and CAT activities (*P* < 0.05), with respective increases of 23.39% and 171.25% under the 1.5% treatment. Conversely, Mn suppressed the POD and CAT activities. Under the 0.25% Mn treatment, POD activity was 895.11 U/g, representing a 42.43% reduction compared to the CK, whereas the decrease in CAT activity was not significant. The co-application of Mn and PE-MPs had no significant effect on POD activity but significantly increased CAT activity (*P* < 0.05), which reached 1108.89 U/g under the 0.50% Mn+1.5% PE-MPs treatment (a 130.83% increase compared to the CK). The addition of PE-MPs increased SOD activity, with a significant increase at the 1.5% level (*P* < 0.05). The addition of Mn significantly reduced SOD activity by 14.83% and 11.64% under the 0.25% and 0.50% treatments, respectively, and the co-application of Mn and PE-MPs also resulted in a decrease in SOD activity. The changes in antioxidant enzyme activities suggested that a 0.25% Mn treatment could mitigate PE-MPs-induced stress in soybeans, thereby exerting a detoxifying effect.

### 3.5 Characteristics of Mn in soybeans under Mn and PE-MPs stress

**Figure 6:**
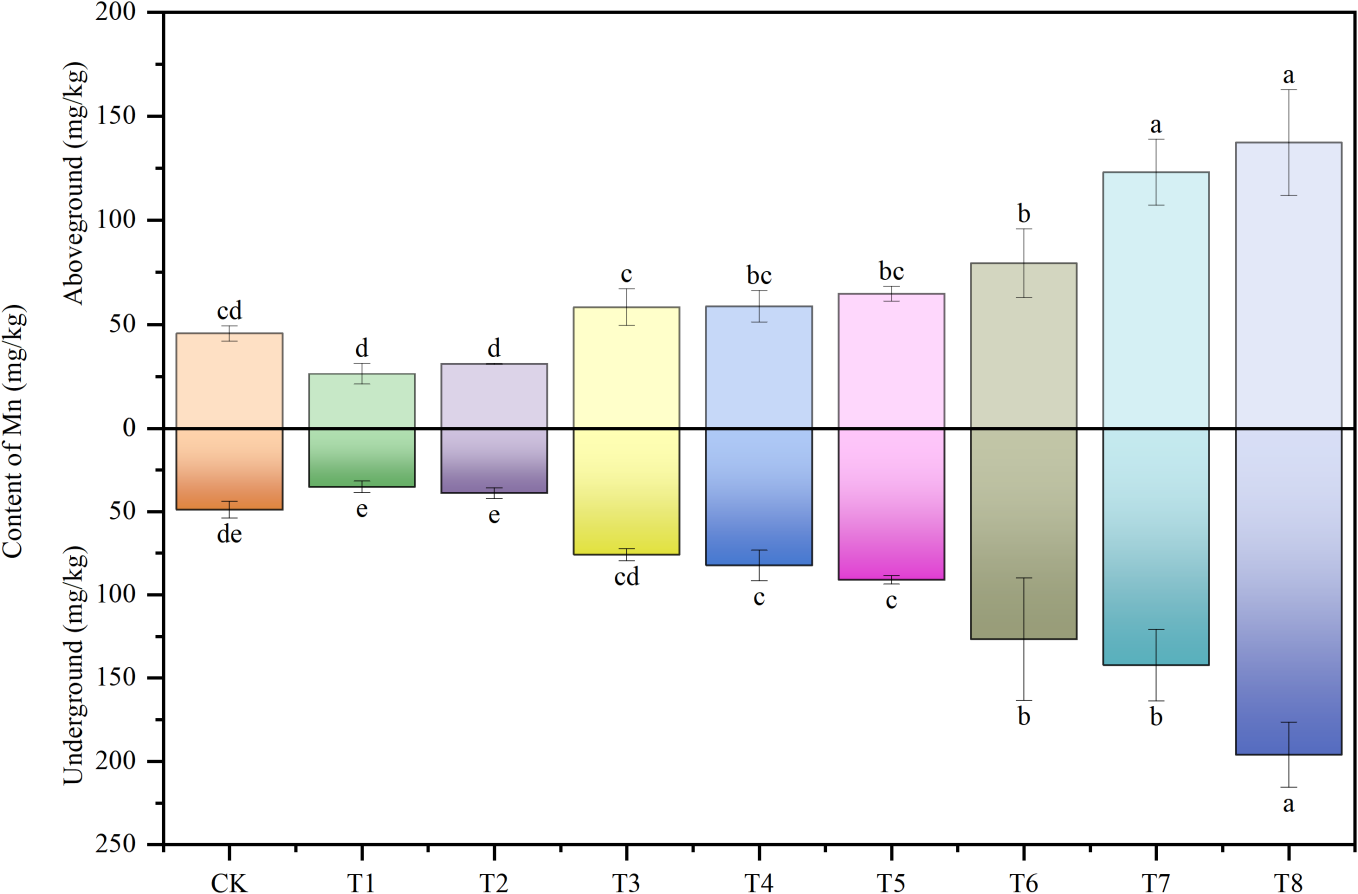
Effects of combined Mn and PE-MPs pollution on the Mn concentration in different parts of the soybean. Different lowercase letters indicate significant differences among the different treatments (*P* < 0.05).

**Figure 6** illustrates the Mn content profiles in the aboveground (stems and leaves) and underground (roots) tissues of soybeans across different treatments. Relative to the control, PE-MPs led to a reduction in Mn content in both aboveground and underground parts, though this decrease was not statistically significant. With increasing Mn addition to the soil, Mn concentrations in both aboveground and underground tissues of soybeans exhibited an upward trend, reaching maximum values of 137.34 mg/kg and 196.01 mg/kg, respectively. The primary factor influencing Mn uptake by soybeans was soil Mn content, whereas the impact of PE-MPs was negligible.

### 3.6 Correlation analysis between soil environmental factors and soybean growth traits

**Figure 7:**
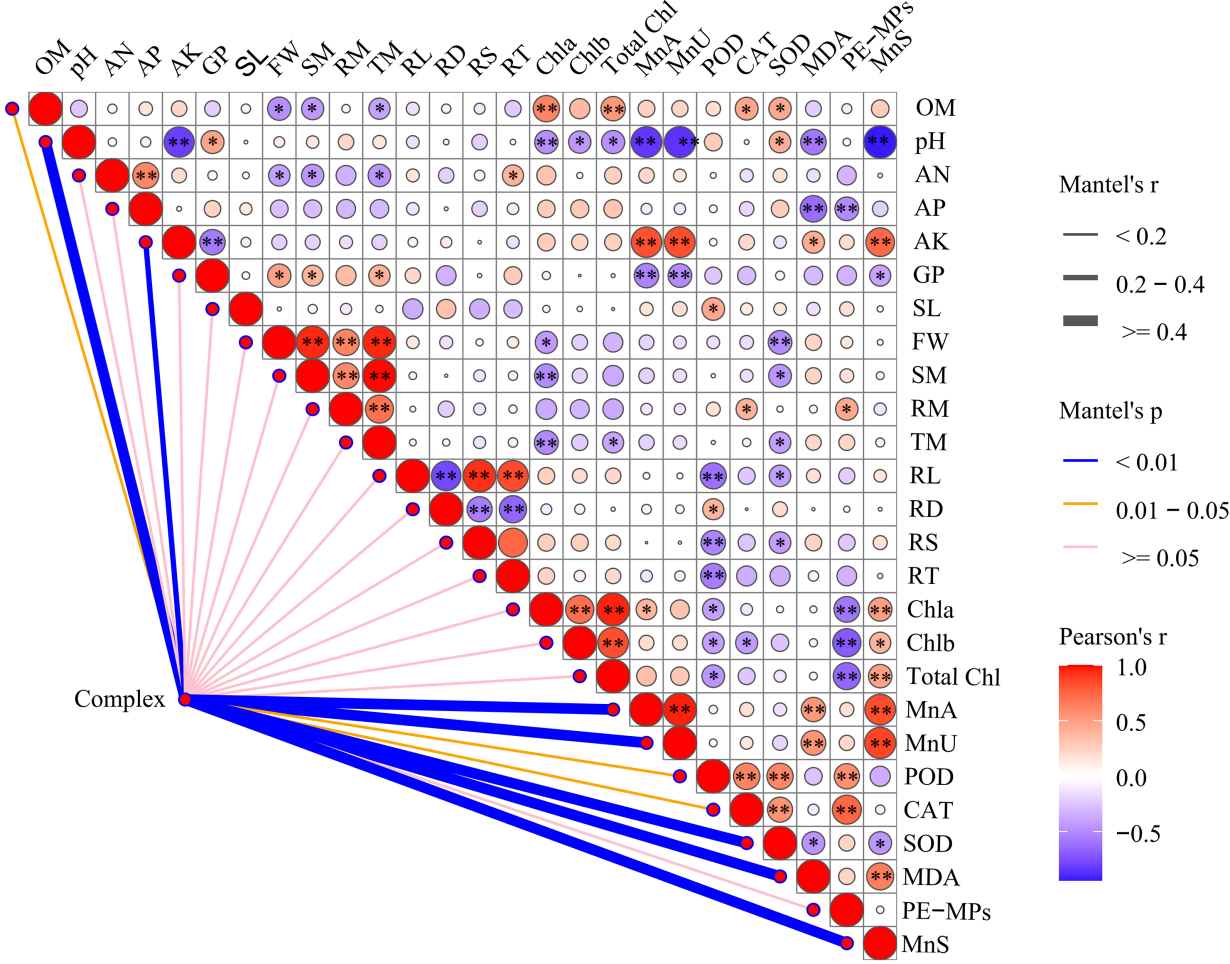
A correlation analysis of the combined pollution factor (Complex), soybean growth parameters, and soil nutrient dynamics. The size of the circle in the matrix denotes the value of the coefficient, with the red circles indicating positive correlations, and the blue circles representing negative correlations. The lines outside the matrix reveal the correlation of the combined pollution and soybean growth parameters, with thicker lines representing more significant correlations. Heat map of the Pearson’s correlation analysis of soybean growth parameters and soil nutrient dynamics. An asterisk (*) denotes a significant difference (*P* < 0.05), while double asterisks (**) indicate a highly significant difference (*P* < 0.01). Complex: combined Mn and PE-MPs pollution, OM: organic matter, AN: available nitrogen, AP: available phosphorus, AK: available potassium, GP: germination percentage, SL: shoot length, FW: fresh weight, SM: shoot biomass, RM: root biomass, TM: total biomass, RL: root length, RD: root diameter, RS: root surface area, RT: root tips, Chla: chlorophyll a, Chlb: chlorophyll b, Total Chl: total chlorophyll, MnA: Mn in the aboveground part, MnU: Mn in underground part, POD: peroxisome, CAT: catalase, SOD: superoxide dismutase, MDA: malondialdehyde, MnS: Mn in soil.

To clarify the main soil environmental factors that influence soybean growth under compound pollution, we used the R software to conduct a Mantel test and generate a correlation heatmap (**Figure 7**). We treated Mn and PE-MPs as a compound pollution factor (referred to as Complex). We analyzed the correlations between the compound pollution factor and the phenotypes of soybeans, as well as the activities of antioxidant enzymes. This analysis aimed to identify the key soil environmental factors governing soybean growth under combined pollution stress. The results revealed that co-exposure to Mn and PE-MPs exerted significant effects (*P* < 0.01) on SOD, MDA, Mn in aboveground parts (MnA), Mn in underground parts (MnU), pH, AK, and Mn in soil (MnS), and significant effects (*P* < 0.05) on CAT, POD, and OM. However, no significant impacts were observed on root morphological traits or growth phenotypes.

In the correlation analysis, PE-MPs had significant negative correlations (*P* < 0.01) with chlorophyll content and AP, alongside significant positive correlations (*P* < 0.01) with POD and CAT. Soil Mn exhibited a significant negative correlation with pH, and significant positive correlations (*P* < 0.01) with AK, chlorophyll content, MDA, and soybean tissue Mn content. Soil OM was significantly negatively correlated (*P* < 0.05) with soybean fresh weight and biomass, and displayed significant positive correlations (*P* < 0.05) with total chlorophyll, CAT, and SOD. Soil pH was significantly negatively correlated (*P* < 0.01) with AK, MnA, MnU, and MnS. Root length was significantly negatively correlated (*P* < 0.01) with root diameter, and significant positive correlations (*P* < 0.01) with root surface area and the number of root tips also observed.

These findings indicate that the combined pollution primarily altered soil pH, OM, and AK, which in turn indirectly affected the soybean POD, CAT, SOD, MDA, and Mn content.

## 4 Discussion

### 4.1 Effects of combined Mn and PE-MPs pollution on soil nutrient regimes

Polyethylene MPs have the potential to significantly decrease the soil pH value (Zhang and Bi, 2025). In this study, PE-MPs exerted no marked influence on the soil. Previous studies have indicated that the influence of MPs on soil pH is contingent upon multiple factors, including the MP type, application rate, and plant root exudates (Qi et al., 2020; Li et al., 2024a). In contrast, combined Mn and PE-MPs pollution significantly lowered soil pH, primarily due to the dissociation of Mn in soil water into Mn^2+^ and SO_4_^2−^ ions. As a weakly acidic cation, Mn^2+^ undergoes hydrolysis with water to release H^+^, directly elevating H^+^ concentrations in the soil solution and thereby reducing pH. This aligns with previous findings that soil application of 800 mg/kg MnSO_4_ induces a significant pH decrease (Qin et al., 2023).

Soil nutrients are primarily derived from the decomposition of soil minerals and OM, supplemented by anthropogenic fertilizer inputs. While some studies have reported that PE-MPs adversely affect the soil carbon content by disturbing the soil carbon cycle (Guo et al., 2023; Huang et al., 2023), our results showed that PE-MPs increased soil OM. This discrepancy can be attributed to two key mechanisms: first, PE-MPs, as a carbonaceous organic material, are detectable by current organic carbon quantification methods, thus contributing directly to the measured OM (Zhang and Bi, 2025); second, the addition of PE-MPs may activate soil organic carbon pools, facilitating the accumulation of soil nutrients into organic carbon fractions (Liu et al., 2017).

In the present study, combined Mn and PE-MPs pollution (at 0.25% MnSO_4_) led to a significant reduction in OM content. This may be due to manganese oxides promoting OM oxidation and carbon mineralization, as reported previously (Trainer et al., 2021; Zhang et al., 2021). Specifically, Mn is likely transformed into manganese dioxide (MnO_2_) in alkaline soils (pH > 7.6), and MnO_2_ enhances OM decomposition. Conversely, at 0.50% MnSO_4_, where the soil pH dropped below 7.5, MnO_2_ precipitation was diminished, resulting in inhibited OM mineralization.

Polyethylene MPs significantly reduced the soil AN content, which was consistent with the findings of Wang et al (Wang et al., 2024c). This suggests that MPs impair soil N availability by suppressing microbial activity (Zang et al., 2020; Lian et al., 2021b). Both Mn and PE-MPs individually decreased soil AP. Although the AP content typically increases with decreasing pH over the range of 7.4–7.8, the observed reduction here may be attributed to the calcareous nature of the soil, which has a high calcium (Ca) content. Under such conditions, Mn^2+^ and Ca^2+^ act synergistically to form Ca-Mn complexes, enhancing P adsorption or precipitation. Additionally, the high adsorption capacity of manganese oxide surfaces enables strong P retention, further reducing its bioavailability (Gao et al., 2020). Under combined Mn and PE-MPs pollution, the soil AK content increased significantly. This was likely due to MnSO_4_-induced pH reduction, which promotes the dissolution of mineral-bound K. Collectively, these results indicate that soil pH plays a pivotal role in mediating the effects of combined Mn and PE-MPs pollution on soil nutrient dynamics.

### 4.2 Effects of combined Mn and PE-MPs pollution on soybean phenotypes

Low Mn concentrations enhanced the soybean germination rate, whereas combined Mn and PE-MPs pollution significantly reduced it. This aligns with previous findings confirming that MPs can markedly inhibit plant seed germination (Li et al., 2021; Shi et al., 2022). Microplastics are known to alter plant morphology and size primarily by reducing height and biomass (Lian et al., 2021a). The combined Mn and PE-MPs pollution had no notable impact on the plant height of soybeans. Biomass peaked under low Mn concentrations, but decreased significantly under high concentrations of Mn and PE-MPs. This suggests that low Mn concentrations have a mitigating effect on the toxicity of PE-MPs.

Root traits, such as root length, root surface area, and average root diameter, are key indicators of root distribution and nutrient uptake capacity (Zhang et al., 2023). Plant roots are the primary accumulation sites for MPs, and MP-induced changes in root traits can directly or indirectly impact plant growth and survival (Bosker et al., 2019; Guo et al., 2022; Xu et al., 2024). Different MP types exert distinct effects on plant roots. Our results showed that PE-MPs reduced the number of root tips, whereas Mn addition resulted in an increase, with no significant effect observed under their co-application. Consistent with studies reporting an increased total root length and surface area in onions grown in soil containing 2% polypropylene MPs (de Souza MaChado et al., 2019; García-Rodríguez Julio et al., 2024), our findings revealed that the MP treatments significantly increased soybean root length. Both Mn and PE-MPs enhanced total root length and surface area in soybeans, with the maximum values observed at low concentrations (0.25% Mn and 0.50% MPs). Additionally, low Mn concentrations significantly reduced the root diameter. Collectively, these results indicate that low Mn concentrations can promote soybean growth and partially mitigate the toxic effects of PE-MPs.

### 4.3 Effects of combined Mn and PE-MPs pollution on the chlorophyll content, MDA levels, and antioxidant enzyme activities in soybeans

Manganese is a critical element for chlorophyll biosynthesis in plants and plays a pivotal role in enhancing photosynthetic efficiency (Wege, 2022). In the present study, the chlorophyll content gradually increased with the addition of Mn. Polyethylene MPs significantly reduced the chlorophyll content. Previous studies have shown that MPs can reduce the chlorophyll content in plants by 15% to 43% (Iswahyudi et al., 2024). Malondialdehyde is a product of lipid peroxidation within cell membranes. It engages in reactions with intracellular biomolecules, including amino acids, unsaturated fatty acids, and proteins. This interaction leads to peroxidative damage of biological membranes, ultimately disrupting their structure and impairing their biological functions. As a well-recognized biochemical marker, the MDA content reflects the extent of membrane lipid peroxidation under environmental stress (Čakar et al., 2021; Sun et al., 2023). Co-exposure to Mn and PE-MPs significantly elevated MDA levels in soybeans. Several studies have found that MPs give rise to a notable elevation in MDA levels within soybeans (Li et al., 2023). Microplastics can exert toxic effects on plant tissues through physical damage or by disrupting antioxidant enzymes. This effect is capable of elevating the H_2_O_2_ level, thereby triggering lipid peroxidation and cell membrane damage (Shi et al., 2022).

Under environmental stresses, plants are capable of generating substantial amounts of reactive oxygen species (ROS), which can inflict damage on plant cells. To counteract this, plants employ an enzymatic defense system comprising SOD, POD, and CAT, which work together to scavenge ROS and protect cells from oxidative damage (Mansoor et al., 2023). To further investigate the impacts of combined Mn and PE-MPs pollution on soybean growth, we analyzed the activities of these antioxidant enzymes. Our results showed that PE-MPs significantly enhanced POD and CAT activities, with increases of 23.39% and 171.25% compared to the CK, respectively. Our results were consistent with the results of a previous study, in which MPs increased the activity of POD by 51% (Azeem et al., 2024). Notably, Mn exposure reduced POD and CAT activities, with a 42.43% decrease in POD activity observed at low Mn concentrations. This indicates that Mn plays a role in alleviating the toxic effects of PE-MPs. Mn is a crucial nutrient element essential for plant growth and development. It exerts its key physiological functions primarily by participating in the construction of the structures of photosynthesis-related proteins and enzymes, facilitating the biosynthesis of growth substances, and regulating gene expression (Frassinetti et al., 2006). Research has indicated that applying 0.1% and 0.2% manganese sulfate in cadmium-contaminated soils can significantly decrease the bioavailability of cadmium (Kanwal et al., 2024). When the Mn content in the soil lies within an appropriate concentration range, it can effectively promote the essential biochemical reactions and physiological processes within plants. This not only contributes to enhancing the nutritional status of plants but also ensures their normal growth and boosts yields (Arif et al., 2016). Furthermore, Mn plays a vital role in scavenging reactive oxygen species (ROS) (He et al., 2021). The findings of this study are consistent with those reported in previous literature.

### 4.4 Key soil environmental factors affecting soybean growth characteristics under combined Mn and MPs pollution

To identify the key soil environmental factors governing soybean growth under combined Mn and MPs pollution, we constructed a correlation heatmap between soil environmental factors and soybean growth indices using the Mantel test. The results indicated that combined Mn and PE-MPs pollution primarily impacted soil pH, OM, and AK. Several previous studies have demonstrated that the presence of MPs typically modifies soil properties, including bulk density, porosity, water-holding capacity, the dissolved OM content, pH, and cation exchange capacity (de Souza MaChado et al., 2019; Zhang et al., 2019; Wang et al., 2022). Microplastics exert indirect effects on plant growth by altering soil properties and functions (Guo et al., 2022). Our findings revealed that the soil pH was highly significantly negatively correlated with root length and root diameter, while exhibiting a highly significant positive correlation with root surface area and the number of root tips. Soil OM displayed a significant negative correlation with soybean fresh weight and biomass. Combined Mn and PE-MPs pollution indirectly influenced soybean POD, CAT, SOD, MDA, and Mn content through its effects on soil factors. In conclusion, combined Mn and PE-MPs pollution exerts significant impacts on soybean growth, albeit through complex underlying mechanisms. Their effects are modulated by multiple factors, including concentration, soil conditions, and soybean cultivars, all of which require more in-depth investigation in future studies.

## 5 Conclusion

This study systematically investigated the effects of PE-MPs, alone or in combination with Mn, on soybean growth through a 30-day pot experiment. The results demonstrated that the addition of Mn and PE-MPs altered soil properties and reduced soil nutrient availability. Specifically, the soil pH decreased significantly, soil OM, AN, and AP also exhibited significant reductions. Notably, single PE-MPs treatments increased soil OM content, whereas combined Mn and PE-MPs application led to a significant decrease in soil OM. Conversely, the AK content increased significantly, exhibiting a highly significant negative correlation with soil pH. Both individual and co-applications of Mn and PE-MPs significantly affected soybean growth. Specifically, individual additions of Mn and PE-MPs enhanced the soybean germination rate, while their co-application reduced it. The co-application of PE-MPs with low Mn concentrations increased soybean biomass. Notably, PE-MPs significantly enhanced the POD, CAT, and SOD activities in soybeans. In contrast, low Mn concentrations significantly decreased the activities of these three enzymes in soybeans. These findings indicate that the addition of low Mn concentrations can alleviate the stress exerted by PE-MPs on soybeans, thereby playing a detoxifying role. Overall, the effects of the combined Mn and PE-MPs pollution on soybean growth primarily influenced the growth characteristics of soybeans by affecting soil properties. At low concentrations, Mn alleviated the stress imposed by polyethylene PE-MPs on soybeans, thereby exerting a detoxifying effect.

This finding holds significant implications for guiding the safe production of soybeans. In farmlands contaminated with microplastics, appropriate modulation of Mn levels may help mitigate ecological toxicity, thereby providing a theoretical basis for soybean cultivation and management in polluted environments. However, this study is subject to certain limitations. First, the experimental duration was relatively short (30 days), which limits the ability to fully assess the long-term and sustained effects of combined Mn and PE-MPs contamination on late-stage soybean growth and yield. Second, the experiment was conducted under controlled pot conditions, which may not adequately reflect the complex interactions among climatic variables, soil microbial communities, and agricultural management practices in real-world field settings. Therefore, future research should aim to elucidate the underlying molecular mechanisms of combined pollution, validate findings through field-based experiments, and extend investigations to include diverse soybean genotypes and more complex multi-pollutant systems, so as to refine risk assessment and regulatory strategies for ensuring agricultural sustainability and food safety.

## Data Availability

The original contributions presented in the study are included in the article/supplementary material. Further inquiries can be directed to the corresponding author.
